# 1-(2-Methyl-6-nitro-4-phenyl-3-quinol­yl)ethanone

**DOI:** 10.1107/S1600536810015473

**Published:** 2010-04-30

**Authors:** Wan-Sin Loh, Hoong-Kun Fun, K. Kiran, S. Sarveswari, V. Vijayakumar

**Affiliations:** a X-ray Crystallography Unit, School of Physics, Universiti Sains Malaysia, 11800 USM, Penang, Malaysia; b Organic Chemistry Division, School of Advanced Sciences, VIT University, Vellore 632 014, India

## Abstract

In the title compound, C_18_H_14_N_2_O_3_, the quinoline ring system is almost planar [maximum deviation = 0.013 (2) Å] and forms a dihedral angle of 60.36 (7)° with the benzene ring. The nitro group is slightly twisted from the attached quinoline ring system, forming a dihedral angle of 9.06 (19)°. In the crystal packing, inter­molecular C—H⋯O hydrogen bonds link the mol­ecules into chains propagating in [010].

## Related literature

For related structures, see: Fun *et al.* (2009 ▶); Loh *et al.* (2009 ▶). For bond-length data, see: Allen *et al.* (1987 ▶). For the stability of the temperature controller used for the data collection, see: Cosier & Glazer (1986 ▶).
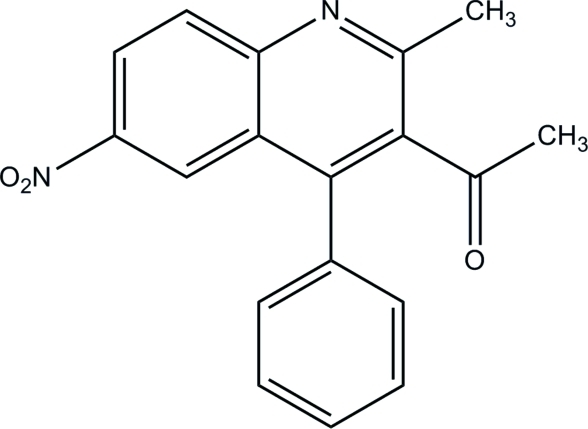


## Experimental

### 

#### Crystal data

C_18_H_14_N_2_O_3_
*M* *_r_* = 306.31
Monoclinic, 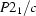

*a* = 13.297 (2) Å
*b* = 7.7689 (12) Å
*c* = 17.9430 (19) Å
β = 129.099 (7)°
*V* = 1438.5 (3) Å^3^
*Z* = 4
Mo *K*α radiation
μ = 0.10 mm^−1^
*T* = 100 K
0.48 × 0.33 × 0.24 mm

#### Data collection

Bruker APEXII DUO CCD area-detector diffractometer
Absorption correction: multi-scan (*SADABS*; Bruker, 2009 ▶) *T*_min_ = 0.954, *T*_max_ = 0.977
14926 measured reflections
4148 independent reflections
3310 reflections with *I* > 2σ(*I*)
*R*_int_ = 0.035

#### Refinement

*R*[*F* ^2^ > 2σ(*F* ^2^)] = 0.061
*wR*(*F* ^2^) = 0.232
*S* = 1.13
4148 reflections
211 parameters
H-atom parameters constrained
Δρ_max_ = 0.86 e Å^−3^
Δρ_min_ = −0.78 e Å^−3^

### 

Data collection: *APEX2* (Bruker, 2009 ▶); cell refinement: *SAINT* (Bruker, 2009 ▶); data reduction: *SAINT*; program(s) used to solve structure: *SHELXTL* (Sheldrick, 2008 ▶); program(s) used to refine structure: *SHELXTL*; molecular graphics: *SHELXTL*; software used to prepare material for publication: *SHELXTL* and *PLATON* (Spek, 2009 ▶).

## Supplementary Material

Crystal structure: contains datablocks global, I. DOI: 10.1107/S1600536810015473/hb5399sup1.cif

Structure factors: contains datablocks I. DOI: 10.1107/S1600536810015473/hb5399Isup2.hkl

Additional supplementary materials:  crystallographic information; 3D view; checkCIF report

## Figures and Tables

**Table 1:** Hydrogen-bond geometry (Å, °)

*D*—H⋯*A*	*D*—H	H⋯*A*	*D*⋯*A*	*D*—H⋯*A*
C3—H3*A*⋯O2^i^	0.93	2.56	3.208 (3)	127

Symmetry code: (i) 
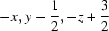
.

**Fig. 1.**
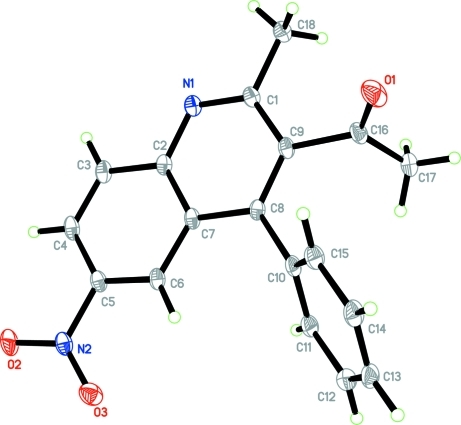
The molecular structure of (I), showing 50% probability displacement ellipsoids.

**Fig. 2.**
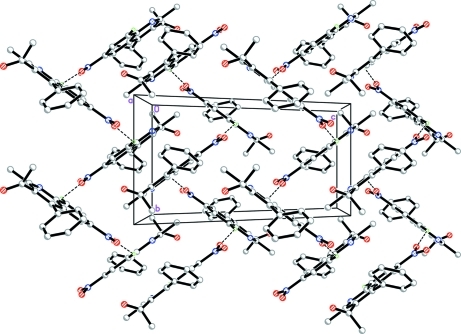
The crystal packing of (I), viewed along the a axis, showing the chains linking down the b axis. H atoms not involved in the intermolecular interactions (dashed lines) have been omitted for clarity.

## supplementary crystallographic information

### Comment

In continuation of our interest in the synthesis and structures of quinolines (Fun *et al.*, 2009; Loh *et al.*, 2009), we now report the title compound, (I).

In the title compound (Fig. 1), the quinoline ring system (C1/N1/C2–C9) is approximately planar with a maximum deviation of 0.013 (2) Å at atom C5. This mean plane of the quinoline ring forms a dihedral angle of 60.36 (7)° with the benzene ring (C10–C15). The nitro group (N2/O2/O3) is slightly twisted from the attached quinoline ring system, forming a dihedral angle of 9.06 (19)°. Bond lengths (Allen *et al.*, 1987) and angles are within normal ranges and are comparable to closely related structures (Fun *et al.*, 2009; Loh *et al.*, 2009).

In the crystal packing (Fig. 2), intermolecular C3—H3A···O2 hydrogen bonds (Table 1) linked the molecules into chains linking down the *b* axis.

### Experimental

A mixture of 5-nitro-2-amino-benzophenone (0.01 M) acetylacetone (0.01 M) and 0.15 ml of concentrated HCl was irradiated under microwave for about 8 min at 240 W. The resultant solid was filtered, dried and purified by column chromatography using 1:1 mixture of ethyl acetate and petroleum ether. *M.P.*: 403 K. Yield: 60%.  Yellow blocks of (I) were recrystallised from chloroform.

### Refinement

All H atoms were positioned geometrically [C–H = 0.93 or 0.96 Å] and were refined using a riding model, with *U*_iso_(H) = 1.2 or 1.5 *U*_eq_(C). A rotating group model was applied to the methyl groups. In the final difference Fourier map, the highest peak and the deepest hole are 1.69 Å and 0.97 Å from atoms H18C and O3, respectively.

### Crystal data

**Table:**

C_18_H_14_N_2_O_3_	*F*(000) = 640
*M**_r_* = 306.31	*D*_x_ = 1.414 Mg m^−^^3^
Monoclinic, *P*2_1_/*c*	Mo *K*α radiation, λ = 0.71073 Å
Hall symbol: -P 2ybc	Cell parameters from 5914 reflections
*a* = 13.297 (2) Å	θ = 3.0–32.9°
*b* = 7.7689 (12) Å	µ = 0.10 mm^−^^1^
*c* = 17.9430 (19) Å	*T* = 100 K
β = 129.099 (7)°	Block, yellow
*V* = 1438.5 (3) Å^3^	0.48 × 0.33 × 0.24 mm
*Z* = 4	

### Data collection

**Table:**

Bruker APEXII DUO CCD area-detector diffractometer	4148 independent reflections
Radiation source: fine-focus sealed tube	3310 reflections with *I* > 2σ(*I*)
graphite	*R*_int_ = 0.035
φ and ω scans	θ_max_ = 30.0°, θ_min_ = 3.0°
Absorption correction: multi-scan (*SADABS*; Bruker, 2009)	*h* = −18→18
*T*_min_ = 0.954, *T*_max_ = 0.977	*k* = −10→10
14926 measured reflections	*l* = −25→24

### Refinement

**Table:**

Refinement on *F*^2^	Primary atom site location: structure-invariant direct methods
Least-squares matrix: full	Secondary atom site location: difference Fourier map
*R*[*F*^2^ > 2σ(*F*^2^)] = 0.061	Hydrogen site location: inferred from neighbouring sites
*wR*(*F*^2^) = 0.232	H-atom parameters constrained
*S* = 1.13	*w* = 1/[σ^2^(*F*_o_^2^) + (0.1525*P*)^2^ + 0.4637*P*] where *P* = (*F*_o_^2^ + 2*F*_c_^2^)/3
4148 reflections	(Δ/σ)_max_ < 0.001
211 parameters	Δρ_max_ = 0.86 e Å^−^^3^
0 restraints	Δρ_min_ = −0.77 e Å^−^^3^

### Special details

**Table:**

Experimental. The crystal was placed in the cold stream of an Oxford Cryosystems Cobra open-flow nitrogen cryostat (Cosier & Glazer, 1986) operating at 100.0 (1) K.
Geometry. All esds (except the esd in the dihedral angle between two l.s. planes) are estimated using the full covariance matrix. The cell esds are taken into account individually in the estimation of esds in distances, angles and torsion angles; correlations between esds in cell parameters are only used when they are defined by crystal symmetry. An approximate (isotropic) treatment of cell esds is used for estimating esds involving l.s. planes.
Refinement. Refinement of *F*^2^ against ALL reflections. The weighted *R*-factor wR and goodness of fit *S* are based on *F*^2^, conventional *R*-factors *R* are based on *F*, with *F* set to zero for negative *F*^2^. The threshold expression of *F*^2^ > σ(*F*^2^) is used only for calculating *R*-factors(gt) etc. and is not relevant to the choice of reflections for refinement. *R*-factors based on *F*^2^ are statistically about twice as large as those based on *F*, and *R*- factors based on ALL data will be even larger.

### Fractional atomic coordinates and isotropic or equivalent isotropic displacement parameters (Å^2^)

**Table:**

	*x*	*y*	*z*	*U*_iso_*/*U*_eq_	
O1	0.22581 (15)	0.7458 (2)	0.39046 (10)	0.0262 (3)	
O2	0.17169 (14)	1.20721 (18)	0.84191 (10)	0.0238 (3)	
O3	0.35548 (14)	1.19938 (18)	0.86969 (10)	0.0229 (3)	
N1	0.00896 (15)	0.73719 (17)	0.49922 (11)	0.0144 (3)	
N2	0.24139 (16)	1.16058 (19)	0.82228 (11)	0.0169 (3)	
C1	0.07078 (17)	0.6966 (2)	0.46604 (12)	0.0142 (3)	
C2	0.07093 (16)	0.8420 (2)	0.57827 (12)	0.0133 (3)	
C3	0.00244 (17)	0.8836 (2)	0.61256 (13)	0.0155 (3)	
H3A	−0.0807	0.8404	0.5809	0.019*	
C4	0.05691 (18)	0.9862 (2)	0.69126 (13)	0.0165 (3)	
H4A	0.0124	1.0125	0.7140	0.020*	
C5	0.18211 (17)	1.0511 (2)	0.73696 (12)	0.0149 (3)	
C6	0.25169 (17)	1.0187 (2)	0.70594 (12)	0.0142 (3)	
H6A	0.3334	1.0667	0.7374	0.017*	
C7	0.19679 (16)	0.9106 (2)	0.62512 (12)	0.0132 (3)	
C8	0.26215 (16)	0.8663 (2)	0.58772 (12)	0.0130 (3)	
C9	0.19839 (17)	0.7586 (2)	0.50918 (12)	0.0141 (3)	
C10	0.39210 (17)	0.9385 (2)	0.62955 (12)	0.0144 (3)	
C11	0.50170 (17)	0.9063 (2)	0.72397 (13)	0.0165 (3)	
H11A	0.4945	0.8404	0.7637	0.020*	
C12	0.62200 (18)	0.9721 (2)	0.75941 (13)	0.0189 (4)	
H12A	0.6946	0.9497	0.8225	0.023*	
C13	0.63368 (18)	1.0716 (2)	0.70039 (14)	0.0190 (4)	
H13A	0.7140	1.1152	0.7240	0.023*	
C14	0.52518 (18)	1.1051 (2)	0.60658 (14)	0.0187 (4)	
H14A	0.5328	1.1721	0.5674	0.022*	
C15	0.40435 (18)	1.0389 (2)	0.57040 (13)	0.0173 (4)	
H15A	0.3320	1.0612	0.5072	0.021*	
C16	0.26170 (18)	0.6954 (2)	0.46785 (13)	0.0180 (4)	
C17	0.3644 (2)	0.5611 (3)	0.52599 (15)	0.0257 (4)	
H17A	0.4068	0.5403	0.4990	0.039*	
H17B	0.3255	0.4564	0.5252	0.039*	
H17C	0.4269	0.6005	0.5910	0.039*	
C18	0.00164 (17)	0.5814 (2)	0.37966 (13)	0.0171 (3)	
H18A	−0.0812	0.5498	0.3610	0.026*	
H18B	0.0523	0.4796	0.3950	0.026*	
H18C	−0.0104	0.6411	0.3277	0.026*	

### Atomic displacement parameters (Å^2^)

**Table:**

	*U*^11^	*U*^22^	*U*^33^	*U*^12^	*U*^13^	*U*^23^
O1	0.0330 (8)	0.0330 (8)	0.0241 (7)	−0.0058 (6)	0.0234 (7)	−0.0039 (6)
O2	0.0298 (8)	0.0284 (7)	0.0265 (7)	0.0043 (6)	0.0240 (7)	−0.0017 (5)
O3	0.0266 (7)	0.0256 (7)	0.0232 (7)	−0.0056 (5)	0.0189 (6)	−0.0054 (5)
N1	0.0177 (7)	0.0126 (6)	0.0182 (7)	0.0002 (5)	0.0139 (6)	0.0014 (5)
N2	0.0234 (8)	0.0166 (7)	0.0188 (7)	0.0026 (5)	0.0172 (6)	0.0017 (5)
C1	0.0183 (8)	0.0125 (7)	0.0168 (8)	−0.0002 (6)	0.0135 (7)	0.0006 (5)
C2	0.0163 (8)	0.0127 (7)	0.0165 (7)	0.0017 (5)	0.0131 (7)	0.0031 (5)
C3	0.0196 (8)	0.0139 (7)	0.0220 (8)	0.0009 (6)	0.0174 (7)	0.0025 (6)
C4	0.0237 (9)	0.0146 (7)	0.0227 (8)	0.0031 (6)	0.0202 (7)	0.0032 (6)
C5	0.0205 (8)	0.0138 (7)	0.0168 (8)	0.0019 (6)	0.0149 (7)	0.0014 (6)
C6	0.0176 (8)	0.0135 (7)	0.0182 (8)	0.0018 (6)	0.0144 (7)	0.0018 (6)
C7	0.0174 (8)	0.0126 (7)	0.0163 (8)	0.0019 (6)	0.0137 (7)	0.0023 (5)
C8	0.0144 (7)	0.0136 (7)	0.0160 (7)	0.0012 (5)	0.0120 (6)	0.0019 (5)
C9	0.0180 (8)	0.0140 (7)	0.0175 (8)	0.0004 (6)	0.0145 (7)	0.0006 (6)
C10	0.0177 (8)	0.0141 (7)	0.0191 (8)	−0.0014 (6)	0.0153 (7)	−0.0026 (6)
C11	0.0198 (8)	0.0175 (7)	0.0184 (8)	0.0002 (6)	0.0151 (7)	−0.0010 (6)
C12	0.0191 (8)	0.0208 (8)	0.0197 (8)	−0.0003 (6)	0.0136 (7)	−0.0020 (6)
C13	0.0191 (8)	0.0188 (8)	0.0280 (9)	−0.0033 (6)	0.0191 (8)	−0.0048 (6)
C14	0.0235 (9)	0.0173 (7)	0.0263 (9)	−0.0019 (6)	0.0209 (8)	−0.0011 (6)
C15	0.0212 (9)	0.0176 (7)	0.0198 (8)	0.0001 (6)	0.0161 (7)	0.0001 (6)
C16	0.0197 (8)	0.0207 (8)	0.0218 (9)	−0.0063 (6)	0.0170 (7)	−0.0076 (6)
C17	0.0224 (9)	0.0314 (10)	0.0269 (10)	0.0037 (7)	0.0173 (8)	−0.0067 (8)
C18	0.0199 (8)	0.0155 (7)	0.0200 (8)	−0.0020 (6)	0.0145 (7)	−0.0024 (6)

### Geometric parameters (Å, °)

**Table:**

O1—C16	1.214 (2)	C9—C16	1.512 (2)
O2—N2	1.2352 (19)	C10—C11	1.394 (3)
O3—N2	1.220 (2)	C10—C15	1.407 (2)
N1—C1	1.322 (2)	C11—C12	1.393 (2)
N1—C2	1.371 (2)	C11—H11A	0.9300
N2—C5	1.472 (2)	C12—C13	1.397 (3)
C1—C9	1.433 (2)	C12—H12A	0.9300
C1—C18	1.500 (2)	C13—C14	1.386 (3)
C2—C7	1.420 (2)	C13—H13A	0.9300
C2—C3	1.421 (2)	C14—C15	1.397 (2)
C3—C4	1.365 (2)	C14—H14A	0.9300
C3—H3A	0.9300	C15—H15A	0.9300
C4—C5	1.406 (2)	C16—C17	1.499 (3)
C4—H4A	0.9300	C17—H17A	0.9600
C5—C6	1.371 (2)	C17—H17B	0.9600
C6—C7	1.416 (2)	C17—H17C	0.9600
C6—H6A	0.9300	C18—H18A	0.9600
C7—C8	1.435 (2)	C18—H18B	0.9600
C8—C9	1.378 (2)	C18—H18C	0.9600
C8—C10	1.493 (2)		
			
C1—N1—C2	117.97 (14)	C11—C10—C8	122.36 (15)
O3—N2—O2	123.74 (15)	C15—C10—C8	118.50 (15)
O3—N2—C5	118.87 (13)	C12—C11—C10	120.56 (16)
O2—N2—C5	117.39 (15)	C12—C11—H11A	119.7
N1—C1—C9	122.51 (15)	C10—C11—H11A	119.7
N1—C1—C18	117.24 (15)	C11—C12—C13	120.11 (17)
C9—C1—C18	120.25 (14)	C11—C12—H12A	119.9
N1—C2—C7	123.38 (14)	C13—C12—H12A	119.9
N1—C2—C3	116.87 (15)	C14—C13—C12	119.78 (17)
C7—C2—C3	119.74 (15)	C14—C13—H13A	120.1
C4—C3—C2	120.92 (16)	C12—C13—H13A	120.1
C4—C3—H3A	119.5	C13—C14—C15	120.43 (16)
C2—C3—H3A	119.5	C13—C14—H14A	119.8
C3—C4—C5	118.26 (14)	C15—C14—H14A	119.8
C3—C4—H4A	120.9	C14—C15—C10	120.00 (17)
C5—C4—H4A	120.9	C14—C15—H15A	120.0
C6—C5—C4	123.46 (16)	C10—C15—H15A	120.0
C6—C5—N2	118.22 (15)	O1—C16—C17	123.29 (16)
C4—C5—N2	118.32 (14)	O1—C16—C9	121.18 (17)
C5—C6—C7	118.74 (16)	C17—C16—C9	115.44 (15)
C5—C6—H6A	120.6	C16—C17—H17A	109.5
C7—C6—H6A	120.6	C16—C17—H17B	109.5
C6—C7—C2	118.85 (14)	H17A—C17—H17B	109.5
C6—C7—C8	123.23 (15)	C16—C17—H17C	109.5
C2—C7—C8	117.91 (15)	H17A—C17—H17C	109.5
C9—C8—C7	117.43 (15)	H17B—C17—H17C	109.5
C9—C8—C10	120.86 (14)	C1—C18—H18A	109.5
C7—C8—C10	121.67 (14)	C1—C18—H18B	109.5
C8—C9—C1	120.78 (14)	H18A—C18—H18B	109.5
C8—C9—C16	121.66 (15)	C1—C18—H18C	109.5
C1—C9—C16	117.51 (14)	H18A—C18—H18C	109.5
C11—C10—C15	119.12 (16)	H18B—C18—H18C	109.5
			
C2—N1—C1—C9	−0.4 (2)	C7—C8—C9—C1	1.1 (2)
C2—N1—C1—C18	179.85 (14)	C10—C8—C9—C1	−176.71 (15)
C1—N1—C2—C7	0.9 (2)	C7—C8—C9—C16	−176.30 (15)
C1—N1—C2—C3	−179.96 (14)	C10—C8—C9—C16	5.9 (2)
N1—C2—C3—C4	179.55 (15)	N1—C1—C9—C8	−0.7 (3)
C7—C2—C3—C4	−1.3 (2)	C18—C1—C9—C8	179.11 (15)
C2—C3—C4—C5	0.6 (2)	N1—C1—C9—C16	176.82 (15)
C3—C4—C5—C6	0.9 (3)	C18—C1—C9—C16	−3.4 (2)
C3—C4—C5—N2	−179.50 (15)	C9—C8—C10—C11	−119.51 (18)
O3—N2—C5—C6	−9.7 (2)	C7—C8—C10—C11	62.8 (2)
O2—N2—C5—C6	170.71 (15)	C9—C8—C10—C15	58.8 (2)
O3—N2—C5—C4	170.68 (15)	C7—C8—C10—C15	−118.90 (17)
O2—N2—C5—C4	−8.9 (2)	C15—C10—C11—C12	−0.2 (2)
C4—C5—C6—C7	−1.7 (3)	C8—C10—C11—C12	178.13 (15)
N2—C5—C6—C7	178.71 (14)	C10—C11—C12—C13	0.1 (3)
C5—C6—C7—C2	0.9 (2)	C11—C12—C13—C14	0.2 (3)
C5—C6—C7—C8	−179.01 (15)	C12—C13—C14—C15	−0.5 (3)
N1—C2—C7—C6	179.57 (15)	C13—C14—C15—C10	0.5 (3)
C3—C2—C7—C6	0.5 (2)	C11—C10—C15—C14	−0.1 (2)
N1—C2—C7—C8	−0.5 (2)	C8—C10—C15—C14	−178.50 (15)
C3—C2—C7—C8	−179.57 (14)	C8—C9—C16—O1	−109.8 (2)
C6—C7—C8—C9	179.40 (15)	C1—C9—C16—O1	72.7 (2)
C2—C7—C8—C9	−0.6 (2)	C8—C9—C16—C17	73.6 (2)
C6—C7—C8—C10	−2.8 (2)	C1—C9—C16—C17	−103.90 (19)
C2—C7—C8—C10	177.24 (14)		

### Hydrogen-bond geometry (Å, °)

**Table:**

*D*—H···*A*	*D*—H	H···*A*	*D*···*A*	*D*—H···*A*
C3—H3A···O2^i^	0.93	2.56	3.208 (3)	127

Symmetry codes: (i) −*x*, *y*−1/2, −*z*+3/2.
